# Levoketoconazole improves clinical signs and symptoms and patient-reported outcomes in patients with Cushing’s syndrome

**DOI:** 10.1007/s11102-020-01103-6

**Published:** 2020-11-20

**Authors:** Eliza B. Geer, Roberto Salvatori, Atanaska Elenkova, Maria Fleseriu, Rosario Pivonello, Przemyslaw Witek, Richard A. Feelders, Marie Bex, Stina W. Borresen, Soraya Puglisi, Beverly M. K. Biller, Fredric Cohen, Francesca Pecori Giraldi

**Affiliations:** 1 Memorial Sloan Kettering Cancer Center, New York, NY USA; 2 Johns Hopkins University, Baltimore, MD USA; 3 Medical University Sofia, Sofia, Bulgaria; 4 Oregon Health and Science University, Portland, OR USA; 5 Università Federico II di Napoli, Naples, Italy; 6 Department of Internal Diseases, Endocrinology and Diabetes, Medical University of Warsaw, Warsaw, Poland; 7 Erasmus Medical Center, Rotterdam, The Netherlands; 8 University Hospitals Leuven, Leuven, Belgium; 9 Department of Medical Endocrinology and Metabolism, Copenhagen University Hospital Rigshospitalet, Copenhagen, Denmark; 10 Department of Clinical and Biological Sciences, San Luigi Gonzaga Hospital, University of Turin, Orbassano, Italy; 11 Massachusetts General Hospital, Boston, MA USA; 12 Strongbridge Biopharma, Trevose, PA USA; 13 Department of Clinical Sciences & Community Health, University of Milan, Milan, Italy; 14 Neuroendocrinology Research Laboratory, Istituto Auxologico Italiano IRCCS, Milan, Italy

**Keywords:** Cushing’s syndrome, Cushing’s disease, Hypercortisolism, Quality of life, Steroidogenesis inhibitor

## Abstract

**Purpose:**

The efficacy of levoketoconazole in treating hypercortisolism was demonstrated in an open-label phase 3 study (SONICS) of adults with endogenous Cushing’s syndrome (CS) and baseline mean urinary free cortisol (mUFC) ≥  1.5× ULN. Clinical signs and symptoms and patient-reported outcomes from the SONICS trial were evaluated in the current manuscript.

**Methods:**

Patients titrated to an individualized therapeutic dose entered a 6-month maintenance phase. Secondary endpoints included investigator-graded clinical signs and symptoms of CS during the maintenance phase, and patient-reported quality of life (CushingQoL questionnaire) and depression symptoms (Beck Depression Inventory II [BDI-II]).

**Results:**

Of 94 enrolled patients, 77 entered the maintenance phase following individualized dose titration. Significant mean improvements from baseline were noted at end of maintenance (Month 6) for acne, hirsutism (females only), and peripheral edema. These improvements were observed as early as Day 1 of maintenance for hirsutism (mean baseline score, 7.8; ∆ − 1.9; *P* < 0.0001), end of Month 1 for acne (mean baseline score, 2.8; ∆ − 1.2; *P* = 0.0481), and Month 4 for peripheral edema (mean baseline score, 1.0; ∆ − 0.5; *P* = 0.0052). Significant mean improvements from baseline were observed by Month 3 of maintenance for CushingQoL (mean baseline score, 44.3; ∆ + 6.9; *P* = 0.0018) and at Month 6 for BDI-II (mean baseline score, 17.1; ∆ − 4.3; *P* = 0.0043) scores. No significant mean improvement was identified in a composite score of 7 other clinical signs and symptoms.

**Conclusions:**

Treatment with levoketoconazole was associated with sustained, meaningful improvements in QoL, depression, and certain clinical signs and symptoms characteristic of CS.

**ClinialTrials.gov identifier**: NCT01838551.

## Introduction

Endogenous Cushing’s syndrome (CS) is a rare and debilitating endocrine disease caused by prolonged elevation in cortisol production [1]. CS is characterized by a variety of clinical signs and symptoms due to hypercortisolism and/or associated hyperandrogenism (in females), including changes in physical appearance (altered fat distribution, hirsutism, acne, skin fragility), metabolic dysregulation (insulin resistance, muscle catabolism), reproductive abnormalities (hypogonadism, menstrual irregularities), and cardiovascular complications (hypertension, atherosclerosis, venous thromboembolism, peripheral edema) [2, 3]. Neuropsychiatric consequences, notably depression, cognitive disturbance, anxiety, and sleep disorders, are also common [4, 5]. As a result of this profound multi-system impact, CS can markedly impair quality of life (QoL), sometimes even after cortisol levels are apparently normalized [6].

Surgery to remove the underlying neoplasia causing excess cortisol production is typically the first-line treatment for CS [7, 8]. Medical therapies, which include pituitary-targeted agents, adrenal steroidogenesis inhibitors, and a glucocorticoid receptor antagonist, are sometimes used as a temporizing measure prior to surgery; when hypercortisolism persists or recurs after surgery; as a bridging treatment until radiotherapy becomes effective; or as primary medical therapy in selected cases [2, 8, 9]. Levoketoconazole, an orally administered ketoconazole stereoisomer, is a potent steroidogenesis inhibitor in development for the treatment of endogenous CS [10]. Results from the phase 3 SONICS (Study of levOketocoNazole In Cushing’s Syndrome) demonstrated that treatment with levoketoconazole normalized mean urinary free cortisol (mUFC) after 6 months of maintenance therapy (without a dose increase during maintenance) in 30% of patients who entered the study, with higher rates of mUFC normalization among those who completed the maintenance phase [11].

Reversal of hypercortisolism has been associated with improvements in clinical signs and symptoms, QoL, and psychological functioning in patients with CS, although residual effects may persist [4, 12]. In SONICS, significant mean improvements were observed from baseline to Month 6 of the maintenance phase in acne, hirsutism (in females), and peripheral edema; QoL was significantly improved and depression was significantly reduced [11]. The current manuscript reports further analysis of the effects of levoketoconazole on investigator-assessed clinical signs and symptoms, patient-reported outcomes, and associated biochemical markers in SONICS.

## Materials and methods

### Study design and patients

SONICS was a multinational, phase 3, single-arm, open-label study of oral levoketoconazole in the treatment of endogenous CS (ClinialTrials.gov: NCT01838551). The study was approved by an institutional review board or independent ethics committee at each site and conducted in accordance with the International Conference on Harmonisation Guideline for Good Clinical Practice and the ethical principles of the Declaration of Helsinki. Detailed study methodology has been published previously [11]. Each patient provided written informed consent to participate in the study.

**Fig. 1:**
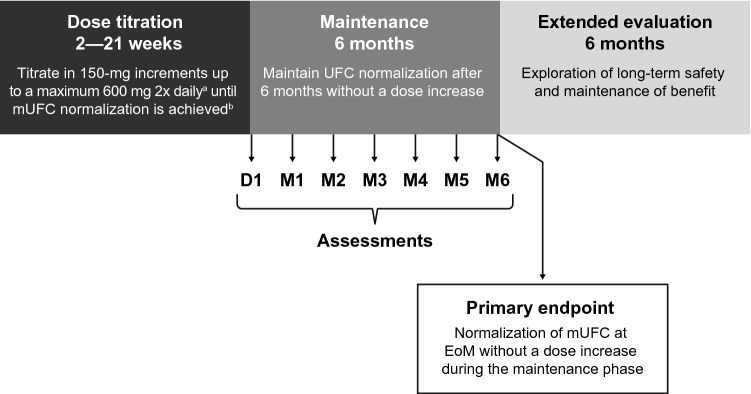
Study design. *D* day, *EoM* end of maintenance, *M* month, *mUFC* mean urinary free cortisol, *UFC* urinary free cortisol, *ULN* upper limit of normal. ^a^All patients started at the protocol-mandated dose of 150 mg twice daily, but a reduction to 150 mg once daily to improve tolerability was allowed. ^b^A therapeutic dose was considered established when 1) mUFC was normalized (i.e., ≤ ULN) or 2) levoketoconazole dose of 600 mg twice daily or a maximal tolerated dose was reached and there was a clinically meaningful partial response in the opinion of the investigator

Adults with a confirmed diagnosis of CS and 24-h mUFC levels ≥ 1.5 × upper limit of normal (ULN) were enrolled in a dose-titration phase (to establish a therapeutic dose) that was followed by a 6-month maintenance phase and a 6-month extended evaluation phase (Fig. 1). During the dose-titration phase, the levoketoconazole dose was adjusted (within the range of 150 mg to 600 mg twice daily) based on mUFC response and tolerability. The levoketoconazole dose was considered therapeutic if (a) mUFC was normalized (i.e., ≤ULN) or (b) a levoketoconazole dose of 600 mg twice daily or a maximal tolerated dose was reached *and* there was a clinically meaningful partial response in the opinion of the investigator. Patients for whom a therapeutic dose was identified were eligible to enter the maintenance phase. Investigators were instructed not to increase the dose of levoketoconazole during the maintenance phase unless it was necessary to maintain cortisol control or in response to safety or tolerability issues.

### Outcomes

The primary endpoint was the percentage of patients who demonstrated mUFC normalization (mUFC ≤ ULN) at the end of the 6-month maintenance phase with no dose increase during maintenance, as previously described [11].

Secondary endpoints included change from baseline to maintenance phase in investigator-assessed clinical signs and symptoms and patient-reported QoL and depression. Clinical signs and symptoms of CS—moon facies, facial plethora, striae, bruising, supraclavicular fat, irregular menstruation (females only), dysmenorrhea (females only), acne, hirsutism (females only), and peripheral edema—were assessed at baseline, at the start of the maintenance phase, and monthly during maintenance therapy. The first 7 items (Cushingoid physical appearance and menstrual status, as listed above) were rated by individual investigators on a scale from 0 to 3, for which 0 = absent, 1 = mild, 2 = moderate, and 3 = severe (total score range, 0–21). As only 5 of these 7 signs and symptoms apply to males, the total score from males was multiplied by 7 and divided by 5 in order to standardize the score for both sexes. Acne was graded by investigators according to Doshi et al. [13]. Hirsutism was evaluated in females according to the rating system described by Hatch et al. [14], as originally described by Ferriman and Gallwey [15]. Peripheral edema was evaluated in 3 locations (lower calf above the medial malleolus, behind the medial malleolus, and dorsum of the foot) and rated according to Brodovicz et al. [16], which was originally described by Seidel et al. [17]. The acne global score ranged from 0 to 44, for which 0 indicated none, 1–18 was mild, 19–30 was moderate, 31–38 was severe, and ≥ 39 was very severe. The hirsutism total score ranged from 0 (none) to 36 (worst). The peripheral edema total score ranged from 0 (none) to 12 (worst).

Quality of life was measured using the CushingQoL questionnaire, a validated 12-item patient-reported measure specific to CS [18]. Patients self-rated each item on a 5-point scale from 1 (“always” or “very much”) to 5 (“never” or “not at all”), and the total score was standardized on a scale from 0 (worst QoL) to 100 (best QoL). Severity of depression was evaluated using the Beck Depression Inventory II (BDI-II), a validated 21-item patient-reported questionnaire [19]. Patients self-rated each item on a scale from 0 to 3 based on how they were feeling during the previous 2 weeks, yielding a total score that ranged from 0 (best) to 63 (worst). BDI-II scores from 0 to 13 indicated no or minimal depression, 14–19 indicated mild depression, 20–28 indicated moderate depression, and 29–63 indicated severe depression [20]. The CushingQoL questionnaire and BDI-II were administered at baseline and Months 3 and 6 of the maintenance phase. The BDI-II was added as an outcome measure after the study was initiated and was administered only to patients who enrolled after the protocol was amended.

Serum testosterone was measured in a central laboratory as total, free, and bioavailable testosterone. Total testosterone was measured using liquid chromatography–tandem mass spectrometry. Free testosterone and bioavailable testosterone (free testosterone + albumin-bound testosterone) were derived from total testosterone and serum albumin and sex hormone binding globulin levels (measured using immunoassay) by the Södergård method [21]. Testosterone levels were analyzed separately for males and females.

### Statistical analysis

The primary endpoint (mUFC normalization without a dose increase during maintenance phase) analysis has been described previously [11]. For the secondary outcomes presented in this paper, analyses included all patients who entered the maintenance phase of the study (the maintenance population). Scores at baseline of the dose-titration phase; Day 1 of the maintenance phase; and after Months 1, 2, 3, 4, 5, and 6 of maintenance (for clinical signs and symptoms and testosterone levels) or Months 3 and 6 of maintenance (for CushingQoL and BDI-II scores) were summarized using descriptive statistics. Mean changes from baseline to each timepoint were analyzed for statistical significance using paired t-tests; no adjustments were made for multiplicity. Post hoc multiple regression analyses evaluated the effect of mUFC value at Month 6 and change from baseline to Month 6 in mUFC level on change from baseline to Month 6 in each of the acne, hirsutism, peripheral edema, CushingQoL, and BDI-II scores separately, adjusting for the respective baseline scores and baseline mUFC. Post hoc multiple regression analyses evaluated the effect of mUFC value at Month 6 on change from baseline to Month 6 in testosterone levels in males and females, separately, adjusting for the baseline levels of testosterone and mUFC.

Exploratory analyses investigated categorical shifts from baseline to Month 6 in measures of CS signs and symptoms, QoL, and depression. For the 7-item scale of Cushingoid physical appearance and menstruation status, the percentage of patients with a categorical shift in severity from baseline to Month 6 (or the last visit in the maintenance phase) was calculated for improvement (in patients with the sign/symptom at baseline [i.e., baseline score ≠ 0]), worsening (in patients with the sign/symptom absent or less than severe at baseline [i.e., baseline score < 3]), and no change. On the CushingQoL questionnaire, a score increase of ≥ 10.1 has been identified as a threshold for clinically meaningful improvement, referred to as a minimally important difference (MID) [22, 23]. For patients with data at baseline and the corresponding study visit, the percentages of patients who met or exceeded the MID threshold were calculated at Months 3 and 6. For the BDI-II score, the percentages of patients with categorical shifts in severity from baseline (minimal, mild, moderate, and severe) were also calculated at Months 3 and 6.

## Results

### Patients

A total of 94 patients enrolled in the study and received ≥ 1 dose of levoketoconazole (intent-to-treat population); 77 patients entered the maintenance phase (maintenance population), and 61 (79%) completed maintenance. The extended evaluation findings are not included in the current analyses, as they were derived from quarterly visits and were considered exploratory rather than secondary outcomes. Baseline characteristics of the maintenance population are shown in Table 1, and baseline scores in Table 2. Mean baseline scores for clinical signs and symptoms were generally consistent with mild symptom severity. However, the mean CushingQoL total score at baseline was 44.3 (median 45.8), ranging up to 94; this was indicative of a population with moderate impairment of health-related QoL and suggested that the patient-reported QoL score captured impairment not reflected by investigator assessments of clinical signs and symptoms. The mean baseline score of 17.1 on the BDI-II was indicative of mild depression [20]. The total daily dose of levoketoconazole at the start of the maintenance phase was 300 mg in 31.2% of patients, 450 mg to 750 mg in 42.9%, and 900 mg to 1200 mg in 26.0%.

**Table 1:** Demographic and baseline characteristics (maintenance population)

Characteristic	Patients (N = 77)
Age, years, mean (SD)	44.1 (12.9)
Female, n (%)	61 (79.2)
Race, n (%)	
White	73 (94.8)
Black	1 (1.3)
Other/unknown	3 (3.9)
BMI, kg/m^2^, mean (SD)	29.9 (7.1)
Time since CS diagnosis, months	
Mean (SD)	65.1 (69.9)
Median (range)	37.5 (0.7–245.4)
Diagnosis of Cushing’s disease, n (%)	67 (87.0)
Treatment-naive for CS, n (%)	23 (29.9)
mUFC^a^ (nmol/24 h)	
Mean (SD)	602.3 (560.4)
Median (range)	410.5 (162.0–3229.5)
mUFC^a^ (mcg/24 h)	
Mean (SD)	218.2 (203.0)
Median (range)	148.7 (58.7–1170.1)
mUFC^a^ (× ULN^b^)	
Mean (SD)	4.4 (4.1)
Median (range)	3.0 (1.2–23.4)^c^

*BMI* body mass index, *CS* Cushing’s syndrome, *mUFC* mean urinary free cortisol, *SD* standard deviation, *UFC* urinary free cortisol, *ULN* upper limit of normal

^a^n = 75; 2 patients did not have ≥ 2 adequate urine samples at baseline

^b^ULN for UFC = 138 nmol/24 h (50 µg/24 h)

^c^One patient with mUFC < 1.5× ULN was excluded because of inadequate urine collection

**Table 2:** Baseline scores on selected secondary outcome measures (maintenance population)

Outcome Measure	Patients (N = 77)		
	n	Mean (SD)	Median (range)
Clinical signs and symptoms			
Acne, global score	75	2.8 (5.8)	0.0 (0–25)
Hirsutism (females), total score	60	7.8 (5.7)	7.0 (0–24)
Peripheral edema, total score	75	1.0 (1.8)	0.0 (0–9)
7 other CSS, total score^a^	61	4.3 (3.8)	3.0 (0–13)
CushingQoL, total score	74	44.3 (21.3)	45.8 (6–94)
BDI-II, total score	59	17.1 (12.9)	14.0 (0–52)

*BDI-II* Beck Depression Inventory II, *CSS* clinical signs and symptoms, *QoL* quality of life, *SD* standard deviation

^a^Moon facies, facial plethora, striae, bruising, supraclavicular fat, irregular menstruation (females only), dysmenorrhea (females only). As only 5 of these 7 signs and symptoms apply to males, their total score was multiplied by 7 and divided by 5 in order to standardize the score for both sexes

### Clinical signs and symptoms of Cushing’s syndrome

**Fig. 2:**
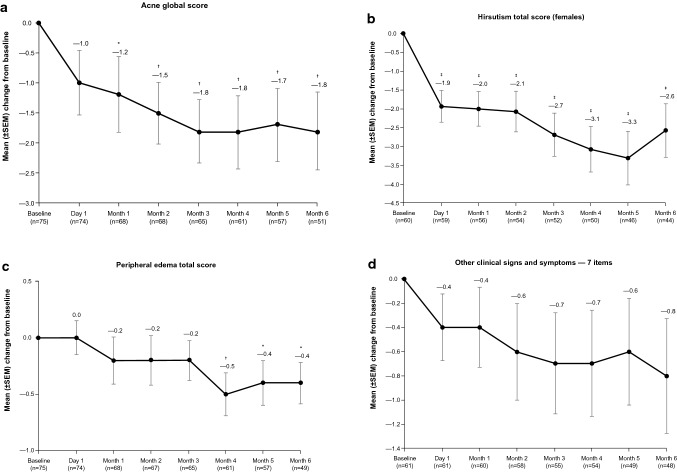
Mean scores for clinical signs and symptoms of Cushing’s syndrome from baseline through the end of the maintenance phase for **a** acne global score, **b** hirsutism total score (females), **c** peripheral edema total score, and **d** total score for 7 other signs and symptoms (maintenance population). Two-sided *P* value from the paired t-test that was performed on the mean change from baseline to each timepoint. Acne global score could range from 0 to 44, where 0 = none, 1–18 = mild, 19–30 = moderate, 31–38 = severe, and ≥39 = very severe. Hirsutism total score could range from 0 (none) to 36 (worst). Peripheral edema total score could range from 0 (none) to 12 (worst). Seven other signs or symptoms (moon facies, facial plethora, striae, bruising, supraclavicular fat, irregular menstruation [females only], dysmenorrhea [females only]) were rated by investigators on a scale from 0 to 3, for which 0 = absent, 1 = mild, 2 = moderate, and 3 = severe (total score range, 0–21). As only 5 of these 7 signs and symptoms apply to males, their total score was multiplied by 7 and divided by 5 in order to standardize the score for both sexes. *SEM* standard error of the mean. *P < 0.05; †*P* < 0.01; ‡*P* < 0.001

Significant mean improvements from baseline to Month 6 were observed in the acne global score, hirsutism total score (females only), and peripheral edema total score. Mean improvement was statistically significant beginning on Day 1 of maintenance (i.e., after the end of dose-titration) for hirsutism (females only; mean change − 1.9; *P* < 0.0001), by the end of Month 1 of maintenance for acne (mean change − 1.2; *P* = 0.0481), and by Month 4 of maintenance for peripheral edema (mean change − 0.5; *P* = 0.0052; Fig. 2). Mean changes from baseline to Month 6 were − 1.8 for acne global score (*P* = 0.0063), − 2.6 for hirsutism total score (females only; *P* = 0.0008), and − 0.4 for peripheral edema total score (*P* = 0.0295). No significant linear relationships between mUFC value or change from baseline at Month 6 and changes from baseline in acne, hirsutism, or peripheral edema scores at Month 6 were observed.

**Fig. 3:**
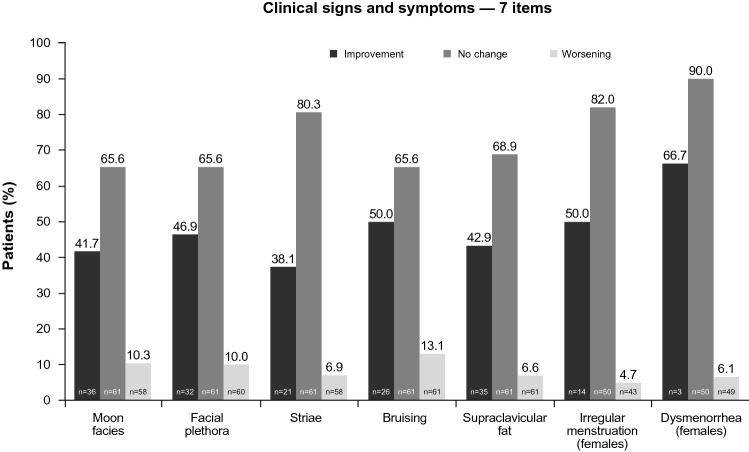
Improvement, no change, or worsening in clinical signs and symptoms of Cushing’s syndrome, based on categorical shift in item score (maintenance population). Improvement was assessed in patients with signs or symptoms at baseline (baseline score ≠ 0) and worsening was assessed in patients with absent or less than severe signs or symptoms at baseline (baseline score < 3). For each item, investigator rating scale was 0 = absent, 1 = mild, 2 = moderate, and 3 = severe

The mean change from baseline of − 0.8 for the total score of 7 items that evaluated Cushingoid physical appearance and menstrual status (moon facies, facial plethora, striae, bruising, supraclavicular fat, irregular menstruation [females only], and dysmenorrhea [females only]) was not statistically significant (*P* = 0.1085). For individual clinical signs and symptoms, improvement from baseline for patients with baseline score ≠ 0 (as determined by categorical shift in item score [0 = “absent” and 3 = “severe”]) was observed in ≥ 38.1% of patients, whereas symptom worsening for those with baseline score < 3 was relatively less common (≤ 13.1% of patients) (Fig. 3).

### Quality of life and depression

**Fig. 4:**
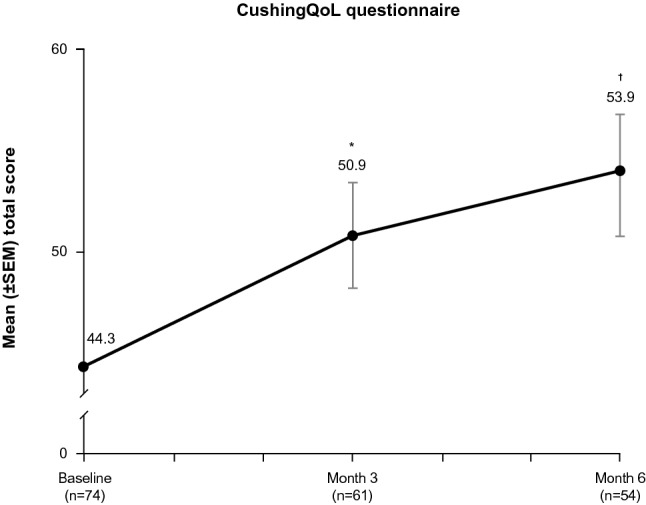
Mean scores from baseline through the end of the maintenance phase for CushingQoL total score (maintenance population). Two-sided *P* value from the paired t-test that was performed on the mean change from baseline to each timepoint. CushingQoL score could range from 0 (worst) to 100 (best). *QoL* quality of life, *SEM* standard error of the mean. *P < 0.01 versus baseline; †*P* < 0.001 versus baseline

Mean total score on the CushingQoL questionnaire increased (improved) from 44.3 at baseline to 50.9 at Month 3 and 53.9 at Month 6 (Fig. 4). Mean changes from baseline in total score were significant at Month 3 (6.9; *P* = 0.0018) and Month 6 (10.6; *P* < 0.0001) assessments. The MID threshold of 10.1 points was met (or exceeded) by 40.0% of individual patients at Month 3 and 47.1% at Month 6 (among the 60 and 51 patients with available data for Month 3 and Month 6, respectively).

**Fig. 5:**
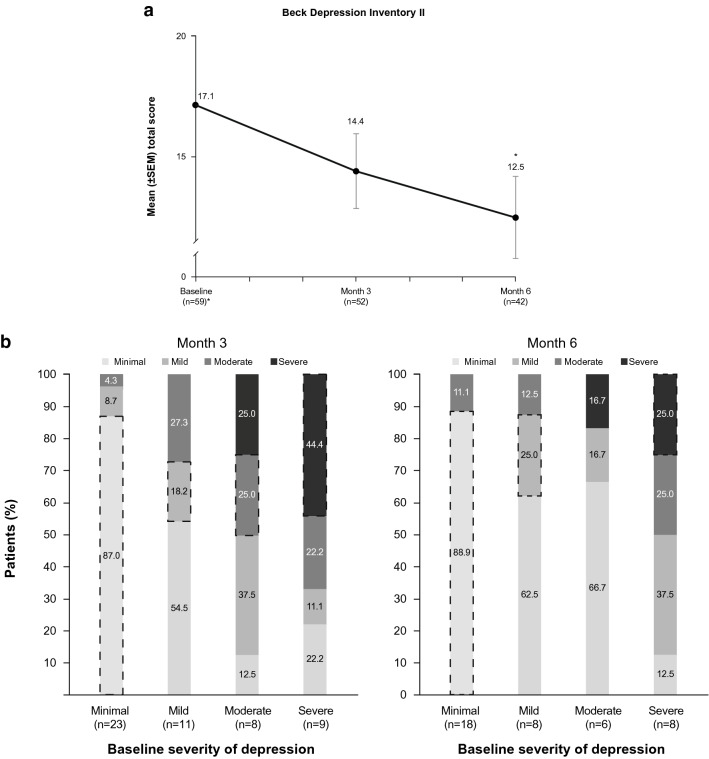
Beck Depression Inventory II total score. **a** Mean scores from baseline through the end of the maintenance phase for BDI-II total score (maintenance population).^a^ Two-sided *P* value from the paired t-test that was performed on the mean change from baseline to each timepoint. BDI-II total score could range from 0 (best) to 63 (worst), with depression severity considered minimal for a score of 0–13, mild for 14–19, moderate for 20–28, and severe for 29–63; thus, a decrease in score reflects clinical improvement. **b** Categorical shifts in BDI-II total score from baseline to Months 3 and 6 of the maintenance phase (maintenance population). Dashed lines identify patient group for which the baseline category was unchanged at postbaseline assessment. *BDI-II* Beck Depression Inventory II, *SEM* standard error of the mean. ^a^As BDI-II was added as an outcome after study initiation, BDI-II data are not available for early recruited patients. *P < 0.01

Mean scores on the BDI-II generally declined (improved) during treatment, from 17.1 at baseline to 14.4 and 12.5 at Months 3 and 6, respectively (Fig. 5a). The mean change from baseline was statistically significant at Month 6 (– 4.3; *P* = 0.0043). In patients with baseline depression severity that was mild, moderate, or severe, categorical shifts in severity reflected improvement in the majority of patients at Month 3 and Month 6 (Fig. 5b).

No significant relationship was found between mUFC value or change from baseline at Month 6 and changes from baseline in CushingQoL or BDI-II scores at Month 6.

### Testosterone levels

**Fig. 6:**
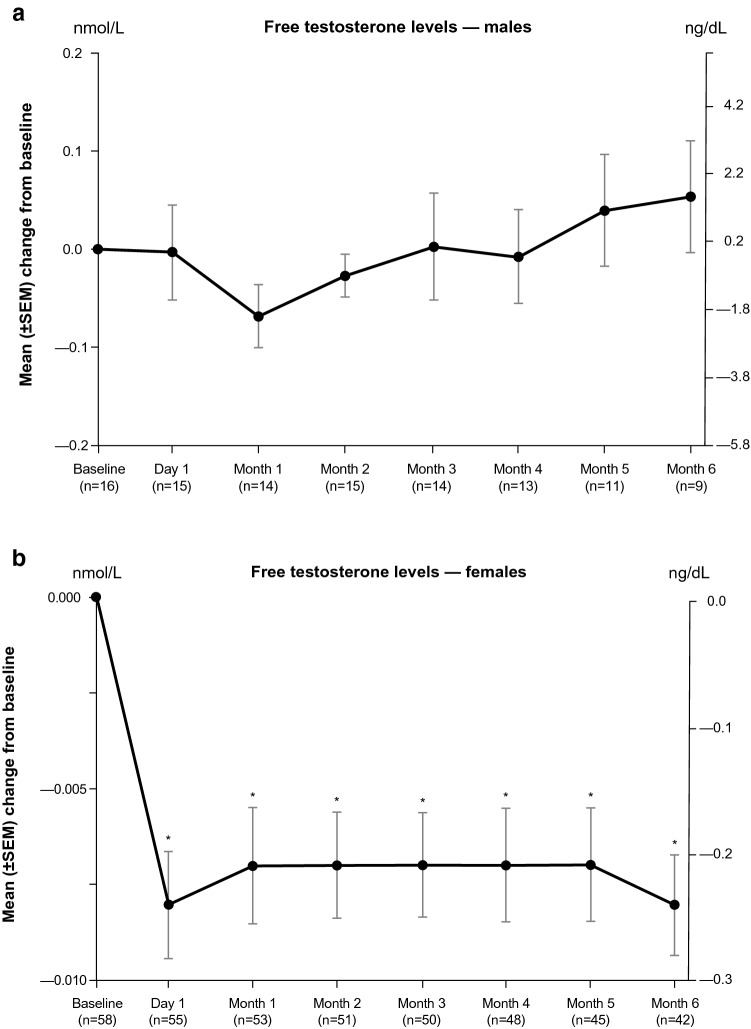
Mean free testosterone levels from baseline through the end of the maintenance phase in **a** males and **b** females (maintenance population). Two-sided *P* value from the paired t-test that was performed on the mean change from baseline to each timepoint. Free testosterone reference levels: males aged 18–69 years, 4.6–22.4 ng/dL (0.160–0.777 nmol/L); males aged 70–89 years, 0.6–7.3 ng/dL (0.021–0.253 nmol/L); females aged 18–69 years, 0.02–0.5 ng/dL (0.0007–0.017 nmol/L); females aged 70–89 years, 0.03–0.5 ng/dL (0.001–0.017 nmol/L). *SEM* standard error of the mean. *P < 0.0001

In males, mean total, bioavailable, and free testosterone increased non-significantly from baseline to Month 6 (Fig. 6; Table 3). In females, reductions in mean total, bioavailable, and free testosterone were observed beginning at Day 1 of the maintenance phase and continued through Month 6 (*P* < 0.0001). There were no significant linear relationships between change in total, bioavailable, and free testosterone levels in males and females and mUFC value at Month 6.

**Table 3:** Testosterone levels from baseline through month 6 (maintenance population)

	Baseline^a^	Month 6^b^	*P* value^c^
Males			
Total testosterone			0.1184
Mean (SD), nmol/L	10.90 (7.91)	22.44 (19.24)	
Mean (SD), ng/dL	314.0 (228.0)	646.7 (554.4)	
Bioavailable testosterone			0.3709
Mean (SD), nmol/L	3.82 (4.07)	4.36 (4.05)	
Mean (SD), ng/dL	110.1 (117.3)	125.5 (116.8)	
Free testosterone			0.3834
Mean (SD), nmol/L	0.18 (0.16)	0.20 (0.17)	
Mean (SD), ng/dL	5.1 (4.7)	5.8 (4.9)	
Females			
Total testosterone			< 0.0001
Mean (SD), nmol/L	0.873 (0.533)	0.518 (0.354)	
Mean (SD), ng/dL	25.2 (15.4)	14.9 (10.2)	
Bioavailable testosterone			< 0.0001
Mean (SD), nmol/L	0.233 (0.192)	0.099 (0.095)	
Mean (SD), ng/dL	6.7 (5.5)	2.9 (2.7)	
Free testosterone			< 0.0001
Mean (SD), nmol/L	0.011 (0.011)	0.004 (0.006)	
Mean (SD), ng/dL	0.32 (0.31)	0.12 (0.16)	

*SD* standard deviation

^a^n = 16 for males and n = 58 for females

^b^n = 9 for males and n = 45 for females

^c^Two-sided *P* value from the paired t-test that was performed on the mean change from baseline to Month 6

## Discussion

SONICS, a large, multinational, phase 3 trial, demonstrated that treatment with levoketoconazole normalized mUFC after 6 months of maintenance therapy, along with improving investigator-graded CS signs and symptoms, patient-reported QoL and depression symptoms, and testosterone levels [11]. In this report, the effects of levoketoconazole on clinical signs and symptoms, patient-reported outcomes, and associated biochemical markers were further evaluated.

The investigator-graded signs and symptoms of CS—acne, hirsutism (in females), and peripheral edema—significantly and consistently improved during 6 months of open-label levoketoconazole maintenance therapy. Improvements in acne and hirsutism were observed early in the maintenance phase, following 2–21 weeks of individualized treatment in the dose-titration phase. Patient-reported QoL significantly improved, and symptoms of depression significantly decreased during treatment with levoketoconazole.

Significant mean reductions in testosterone levels were noted in females, likely accounting for the investigator-assessed improvements noted in acne and hirsutism. Interestingly, testosterone did not decline in males, but rather trended toward an increase on average. The reduction of testosterone in females also has been seen with ketoconazole treatment in patients with CS [24–26]. Females with active CS typically experience hyperandrogenism as a result of stimulated adrenal androgen production, which can be suppressed by ketoconazole, an inhibitor of CYP17A1, and other enzymes in the androgen synthesis pathway [10, 27]. On the other hand, males with active CS tend to experience reduced testosterone production due to glucocorticoid-induced gonadotropin suppression and subsequent reduction in testicular testosterone production [27, 28]. As ketoconazole tends to suppress testosterone production in males with a normal hypothalamus-pituitary-gonadal axis [29, 30], one might expect that it would also suppress testosterone in CS. However, case studies suggest that ketoconazole may improve or not change serum testosterone in males with CS [31, 32], perhaps via improvement of gonadotropin secretion that, in turn, stimulates testicular testosterone synthesis. The current study suggests that levoketoconazole is similar to ketoconazole in this regard, without any negative (and, possibly, with positive) effects on gonadal function in men.

In contrast to the significant improvements in acne, hirsutism, and peripheral edema scores, a 7-item composite score of Cushingoid appearance and menstruation status that included moon facies, facial plethora, striae, bruising, supraclavicular fat, and irregular menstruation and dysmenorrhea, which indicated only mild abnormality at baseline, decreased, but not significantly, during treatment. To be nominally significant (at *P* < 0.05) at Month 6, a mean decrease in the total score of − 0.94, representing a relative reduction in the mean score of 22%, would have been required, whereas a reduction of − 0.8 was observed. As a minimal clinically important difference in this score has not been established, it is unknown whether the reduction from baseline in the mean score noted in the present study is of potential clinical relevance. The fact that photographs were not taken at every visit may have limited the investigators’ ability to identify changes in patients’ physical appearance over time.

Patient-reported symptoms of depression, which were mild at baseline, nonetheless improved with therapy, and QoL, which was moderately impaired at baseline, substantially improved. Notably, the improvements in patient-reported and physician-assessed outcomes were observed in the context of substantial mean reductions in mUFC levels. In 55 maintenance phase completers with UFC data, mUFC normalized at Month 6 in 34 patients (62%), and 43 patients (78%) experienced a decrease of at least 50% (or normalization) in mUFC [11]. However, no linear correlation was found between improvement in clinical signs and symptoms (acne, hirsutism, peripheral edema), CushingQoL, or BDI-II scores and changes in or absolute values of mUFC from baseline to Month 6. This finding is similar to a prior prospective multinational study of pasireotide in Cushing’s disease that demonstrated a lack of correlation between baseline UFC levels and baseline severity of a large number of features of clinical hypercortisolism [33]. It may be the case that the severity of CS clinical features is more strongly associated with the duration of hypercortisolism than the UFC per se. Consistent with this hypothesis, a significant correlation was reported between change in CushingQoL score and change in mUFC levels after 12 months, but not after 6 months, of treatment with pasireotide [22]. Therefore, although the average duration of therapy at the end of Month 6 of the maintenance phase in the SONICS study was approximately 230 days [11], it may not have been sufficiently long to observe a correlation between improvement in clinical features of CS and mUFC levels.

The CushingQoL questionnaire includes items specific to the disease (e.g., “I’m worried about the changes in my physical appearance”), as well as more general health-related QoL items (e.g., “My illness affects my everyday activities”) [18]. The mean baseline score, which indicated moderate QoL impairment, appears to indicate that the impact of the disease on patient perceptions of illness is not captured by the investigator assessments of signs and symptoms, at least with the instruments used in this study. This issue deserves further research, as a recently published study of acromegaly, another rare pituitary disease, suggests that there is poor concordance between physician and patient perceptions of disease and treatment impact on the frequency and severity of symptoms [34]. Nevertheless, whether similar discordance exists in the medically treated CS population remains unknown.

Depressive symptoms were not severe at baseline, as indicated by the mean BDI-II score; yet, a statistically significant mean decrease was observed at Month 6 of the maintenance phase. Similar baseline impairment and degree of improvement in BDI-II score with medical therapies for CS were noted in prospective studies of mifepristone [35] and pasireotide [36], but to our knowledge this is the first published prospective phase 3 study of an adrenal steroidogenesis inhibitor that assessed this particular outcome [11].

In SONICS, the most commonly reported adverse events during the dose-titration and maintenance phases were nausea (32% of patients) and headache (28% of patients) [11]. QT interval prolongation, identified as an adverse event in 5 patients (5%), was reversible with temporary drug discontinuation. Reversible elevations > 3 × ULN of alanine aminotransferase were observed at least once during the maintenance phase in 11% of patients. In a separate analysis of metabolic parameters from SONICS data, significant mean improvement from baseline to Month 6 was observed in biomarkers of cardiovascular risk, both in the overall patient population [11] and in patients with comorbid type 2 diabetes [37].

The longitudinal design and the large number of patients in SONICS are study strengths that allow evaluation of improvements over the course of treatment with levoketoconazole. The main limitations of the study are the use of open-label treatment (both participants and investigators aware), the lack of a placebo arm, and unblinded study assessments. Knowledge of study treatment and assessments made by unblinded assessors are potentially important sources of bias, particularly with regard to subjective assessments, such as patient-reported QoL and depression symptom inventory score. In addition, because of the disproportionate representation of etiologies of CS among patients in this study (67 patients with Cushing’s disease and 6 patients with adrenal-dependent CS in the maintenance population), a correlation analysis between changes in signs and symptoms and the type of CS was unlikely to provide meaningful information and therefore was not undertaken.

In conclusion, levoketoconazole provides sustained improvements in relevant investigator-assessed signs and symptoms and patient-reported QoL and depression outcomes for patients with CS; these improvements are associated with changes in biochemical disease markers including serum testosterone and mUFC levels.

## Data Availability

Data are available upon request.

## References

[1] Sharma ST, Nieman LK, Feelders RA (2015). Cushing’s syndrome: epidemiology and developments in disease management. Clin Epidemiol.

[2] Feelders RA, Newell-Price J, Pivonello R, Nieman LK, Hofland LJ, Lacroix A (2019). Advances in the medical treatment of Cushing’s syndrome. Lancet Diabetes Endocrinol.

[3] Pivonello R, Isidori AM, De Martino MC, Newell-Price J, Biller BM, Colao A (2016). Complications of Cushing’s syndrome: state of the art. Lancet Diabetes Endocrinol.

[4] Pivonello R, Simeoli C, De Martino MC, Cozzolino A, De Leo M, Iacuaniello D, Pivonello C, Negri M, Pellecchia MT, Iasevoli F, Colao A (2015). Neuropsychiatric disorders in Cushing’s syndrome. Front Neurosci.

[5] Santos A, Resmini E, Pascual JC, Crespo I, Webb SM (2017). Psychiatric symptoms in patients with Cushing’s syndrome: prevalence, diagnosis and management. Drugs.

[6] Broersen LHA, Andela CD, Dekkers OM, Pereira AM, Biermasz NR (2019). Improvement but no normalization of quality of life and cognitive functioning after treatment for Cushing’s syndrome. J Clin Endocrinol Metab.

[7] Biller BM, Grossman AB, Stewart PM, Melmed S, Bertagna X, Bertherat J, Buchfelder M, Colao A, Hermus AR, Hofland LJ, Klibanski A, Lacroix A, Lindsay JR, Newell-Price J, Nieman LK, Petersenn S, Sonino N, Stalla GK, Swearingen B, Vance ML, Wass JA, Boscaro M (2008). Treatment of adrenocorticotropin-dependent Cushing’s syndrome: a consensus statement. J Clin Endocrinol Metab.

[8] Pivonello R, De Leo M, Cozzolino A, Colao A (2015). The treatment of Cushing’s disease. Endocr Rev.

[9] Tritos NA, Biller BM (2018). Medical therapy for Cushing’s syndrome in the twenty-first century. Endocrinol Metab Clin North Am.

[10] Fleseriu M, Castinetti F (2016). Updates on the role of adrenal steroidogenesis inhibitors in Cushing’s syndrome: a focus on novel therapies. Pituitary.

[11] Fleseriu M, Pivonello R, Elenkova A, Salvatori R, Auchus RJ, Feelders RA, Geer EB, Greenman Y, Witek P, Cohen F, Biller BM (2019). Efficacy and safety of levoketoconazole in the treatment of endogenous Cushing’s syndrome (SONICS): a phase 3, multicentre, open-label, single-arm trial. Lancet Diabetes Endocrinol.

[12] Broersen LHA, Jha M, Biermasz NR, Pereira AM, Dekkers OM (2018). Effectiveness of medical treatment for Cushing’s syndrome: a systematic review and meta-analysis. Pituitary.

[13] Doshi A, Zaheer A, Stiller MJ (1997). A comparison of current acne grading systems and proposal of a novel system. Int J Dermatol.

[14] Hatch R, Rosenfield RL, Kim MH, Tredway D (1981). Hirsutism: implications, etiology, and management. Am J Obstet Gynecol.

[15] Ferriman D, Gallwey JD (1961). Clinical assessment of body hair growth in women. J Clin Endocrinol Metab.

[16] Brodovicz KG, McNaughton K, Uemura N, Meininger G, Girman CJ, Yale SH (2009). Reliability and feasibility of methods to quantitatively assess peripheral edema. Clin Med Res.

[17] Seidel HM, Ball JW, Dains JE, Benedict GW, Schrefer S (1995). Heart and blood vessels. Mosby’s guide to physical examination.

[18] Webb SM, Badia X, Barahona MJ, Colao A, Strasburger CJ, Tabarin A, van Aken MO, Pivonello R, Stalla G, Lamberts SW, Glusman JE (2008). Evaluation of health-related quality of life in patients with Cushing’s syndrome with a new questionnaire. Eur J Endocrinol.

[19] Beck AT, Steer RA, Ball R, Ranieri W (1996). Comparison of beck depression inventories -IA and -II in psychiatric outpatients. J Pers Assess.

[20] Beck AT, Steer RA, Brown GK (1996). Beck Depression Inventory®–II (BDI®–II).

[21] Södergård R, Bäckström T, Shanbhag V, Carstensen H (1982). Calculation of free and bound fractions of testosterone and estradiol-17 beta to human plasma proteins at body temperature. J Steroid Biochem.

[22] Webb SM, Ware JE, Forsythe A, Yang M, Badia X, Nelson LM, Signorovitch JE, McLeod L, Maldonado M, Zgliczynski W, de Block C, Portocarrero-Ortiz L, Gadelha M (2014). Treatment effectiveness of pasireotide on health-related quality of life in patients with Cushing’s disease. Eur J Endocrinol.

[23] Nelson LM, Forsythe A, McLeod L, Pulgar S, Maldonado M, Coles T, Zhang Y, Webb SM, Badia X (2013). Psychometric evaluation of the Cushing’s Quality-of-Life questionnaire. Patient.

[24] Loli P, Berselli ME, Tagliaferri M (1986). Use of ketoconazole in the treatment of Cushing’s syndrome. Clin Endocrinol Metab.

[25] Weber MM, Luppa P, Engelhardt D (1989). Inhibition of human adrenal androgen secretion by ketoconazole. Klin Wochenschr.

[26] Sonino N, Boscaro M, Paoletta A, Mantero F, Ziliotto D (1991). Ketoconazole treatment in Cushing’s syndrome: experience in 34 patients. Clin Endocrinol (Oxf).

[27] Vierhapper H, Nowotny P, Waldhäusl W (2000). Production rates of testosterone in patients with Cushing’s syndrome. Metabolism.

[28] Luton J-P, Thieblot P, Valcke J-C, Mahoudeau JA, Bricaire H (1977). Reversible gonadotropin deficiency in male Cushing’s disease. J Clin Endocrinol Metab.

[29] Santen RJ, Van den Bossche H, Symoens J, Brugmans J, DeCoster R (1983). Site of action of low dose ketoconazole on androgen biosynthesis in men. J Clin Endocrinol Metab.

[30] Pont A, Williams PL, Azhar S, Reitz RE, Bochra C, Smith ER, Stevens DA (1982). Ketoconazole blocks testosterone synthesis. Arch Intern Med.

[31] Mortimer RH, Cannell GR, Thew CM, Galligan JP (1991). Ketoconazole and plasma and urine steroid levels in Cushing’s disease. Clin Exp Pharmacol Physiol.

[32] De Martin M, Toja PM, Goulene K, Radaelli P, Cavagnini F, Stramba-Badiale M, Pecori Giraldi F (2016). No untoward effect of long-term ketoconazole administration on electrocardiographic QT interval in patients with Cushing’s disease. Basic Clin Pharmacol Toxicol.

[33] Petersenn S, Newell-Price J, Findling JW, Gu F, Maldonado M, Sen K, Salgado LR, Colao A, Biller BM, Pasireotide BSG (2014). High variability in baseline urinary free cortisol values in patients with Cushing’s disease. Clin Endocrinol (Oxf).

[34] Geer EB, Sisco J, Adelman DT, Ludlam WH, Haviv A, Gelbaum D, Liu S, Mathias SD, Shi L (2020). Observed discordance between outcomes reported by acromegaly patients and their treating endocrinology medical provider. Pituitary.

[35] Fleseriu M, Biller BM, Findling JW, Molitch ME, Schteingart DE, Gross C, SEISMIC Study Investigators (2012). Mifepristone, a glucocorticoid receptor antagonist, produces clinical and metabolic benefits in patients with Cushing’s syndrome. J Clin Endocrinol Metab.

[36] Pivonello R, Petersenn S, Newell-Price J, Findling JW, Gu F, Maldonado M, Trovato A, Hughes G, Salgado LR, Lacroix A, Schopohl J, Biller BM, Pasireotide BSG (2014). Pasireotide treatment significantly improves clinical signs and symptoms in patients with Cushing’s disease: results from a Phase III study. Clin Endocrinol (Oxf).

[37] Fleseriu M, Pivonello R, Elenkova A, Salvatori R, Auchus RJ, Feelders RA, Geer EB, Greenman Y, Witek P, Cohen F, Biller BMK (2019) Results from the phase 3 multicenter SONICS study of levoketoconazole: subgroup analysis of Cushing’s syndrome patients with diabetes mellitus. In: ECE 2019: 21st European Congress of Endocrinology, Lyon, France, May 18–21 2019

