# Correction to: Levoketoconazole improves clinical signs and symptoms and patient-reported outcomes in patients with Cushing’s syndrome

**DOI:** 10.1007/s11102-020-01116-1

**Published:** 2020-12-14

**Authors:** Eliza B. Geer, Roberto Salvatori, Atanaska Elenkova, Maria Fleseriu, Rosario Pivonello, Przemyslaw Witek, Richard A. Feelders, Marie Bex, Stina W. Borresen, Soraya Puglisi, Beverly M. K. Biller, Fredric Cohen, Francesca Pecori Giraldi

**Affiliations:** 1 Memorial Sloan Kettering Cancer Center, New York, NY USA; 2 Johns Hopkins University, Baltimore, MD USA; 3 Medical University Sofia, Sofia, Bulgaria; 4 Oregon Health and Science University, Portland, OR USA; 5 Università Federico II di Napoli, Naples, Italy; 6 Department of Internal Diseases, Endocrinology and Diabetes, Medical University of Warsaw, Warsaw, Poland; 7 Erasmus Medical Center, Rotterdam, The Netherlands; 8 University Hospitals Leuven, Leuven, Belgium; 9 Department of Medical Endocrinology and Metabolism, Copenhagen University Hospital Rigshospitalet, Copenhagen, Denmark; 10 Department of Clinical and Biological Sciences, San Luigi Gonzaga Hospital, University of Turin, Orbassano, Italy; 11 Massachusetts General Hospital, Boston, MA USA; 12 Strongbridge Biopharma, Trevose, PA USA; 13 Department of Clinical Sciences & Community Health, University of Milan, Milan, Italy; 14 Neuroendocrinology Research Laboratory, Istituto Auxologico Italiano IRCCS, Milan, Italy

## Correction to: Pituitary 10.1007/s11102-020-01103-6

The original version of the article unfortunately contained an error in the first name and the surname of one of the authors in the author group. The last author name was incorrectly published as ‘F. Pecori Giraldi’ and the corrected name is ‘Francesca Pecori Giraldi’ (First name: Francesca; Surname: Pecori Giraldi).

The original article has been corrected.

